# Determining Policy Targets for Reducing the Number of Stunted Papuan Children Under Five Years Old in Indonesia: A Secondary Data Analysis of the 2022 Indonesian National Nutritional Status Survey

**DOI:** 10.21315/mjms-10-2024-798

**Published:** 2025-06-30

**Authors:** Ratna Dwi Wulandari, Agung Dwi Laksono, Sarni Rante Allo Bela, Mona Safitri Fatiah, Nikmatur Rohmah, Noor Edi Widya Sukoco, Nurhasmadiar Nandini, Novia Luthviatin

**Affiliations:** 1 Faculty of Public Health, Universitas Airlangga, Surabaya, East Java, Indonesia; 2 National Research and Innovation Agency Republic of Indonesia, Jakarta, Indonesia; 3 Faculty of Public Health, Universitas Cendrawasih, Jayapura, Papua, Indonesia; 4 Faculty of Health Science, Muhammadiyah University of Jember, East Java, Indonesia; 5 Faculty of Public Health, Universitas Diponegoro, Semarang, Central Java, Indonesia; 6 Faculty of Public Health, Universitas Jember, Jember, East Java, Indonesia

**Keywords:** stunting, under five years old, Papuan, nutritional status, public health nutrition

## Abstract

**Background:**

Although stunting continues to decline, it remains a problem in Indonesia, particularly in underdeveloped areas such as the Papua region. This study determined policy targets for reducing the number of stunted Papuan children under five years old in Indonesia.

**Methods:**

This secondary analysis focused on the 2022 Indonesian National Nutritional Status Survey data. A total of 13,268 individuals under five years old were studied. Eleven independent variables were examined: province, domicile, mother’s age, education, marital status, work, wealth, antenatal care (ANC), children’s age, sex, early initiation of breastfeeding (EIBF), and nutritional status. A binary logistic regression test was employed for all examinations.

**Results:**

The prevalence of Papuan toddler stunting was 29.8%. Two factors related to residence were classified into five types: province and residence. Four maternal demographic characteristics were associated with stunting in Papua under five years old: age, education, marital status, and employment status. All wealth statuses were more likely than the richest to be stunted under five years old. Papuan mothers without ANC were 1.150 times more likely to have stunted children under five years old (95% CI: 1.116–1.185). All children were more likely to be stunted than those aged 0–1 months. Boys were 1.318 times more likely to be stunted than girls (95% CI: 1.297–1.340). Five patients without EIBF were 1.092 times more likely to be stunted than those with EIBF (95% CI: 1.060–1.124).

**Conclusion:**

Eleven factors were related to stunting in Papuan children: province, residence, maternal age, education, marital status, employment, wealth, ANC, age, sex, and EIBF.

## Introduction

Stunting is a condition of growth failure that results from nutritional deficiencies and recurrent infections during pregnancy, extending up to the first 1,000 days of life. This condition becomes apparent when a child reaches the age of two years (1) and shows a strong correlation with perinatal conditions. Stunting is characterised by measuring a child’s length (lying down for children under two years old), height (standing for children over two years old), or weight compared to international growth standards, known as Z-score values. If the Z-score calculation equals the median value, it is “0.” A positive Z-score was above the median, whereas a negative Z-score was below the median (2).

According to data from the World Health Organization, the estimated proportion of stunting incidents in children under five years old in 2022 is approximately 22.3% globally. This estimate indicates a decrease compared to previous years, specifically from 22.7% in 2020 to 22.5% in 2021 and further to 22.3% in 2022, representing a decline of approximately 0.2% each year (3). Stunting among children under five years old is widespread across various WHO intervention regions worldwide, with Southeast Asia holding the second-highest position at around 30.1%, followed by Africa at 31.0% (3). Reducing stunting among children under five years old aligns with the focus of programmes outlined in Sustainable Development Goal Target 2.2. It is also part of the WHO target for 2025, which aims for a 40% reduction in stunting incidents in five fields (2, 4). Given these objectives, collaborative efforts across sectors are essential to achieve the set of Sustainable Development Goals and targets.

The incidence of stunting in developing countries, including Indonesia, which is a member of Southeast Asia, is notable. In 2022, the estimated proportion of stunting incidents in Indonesia among SEA member countries was 31.0%, ranking Indonesia as the third highest, followed by Timor-Leste (45.1%) and India (31.7%) (5). In Indonesia, the proportion of stunting incidents among children under five years old from 2019 to 2022 experienced a decline of 3.3% from 2019 (27.7%) to 2021 (24.4%) and a further decrease of 2.8% from 2021 (24.2%) to 2022 (21.6%) (6). While this is positive news for Indonesia, it is also a cause for concern, as the proportion is still far from the national target of reducing stunting incidents to 14% by 2024.

Stunting incidents occur within the first 1,000 days of life and, if not addressed promptly, can have long-term negative impacts on children, such as loss of productivity, lower future income, and increased risk of chronic diseases (7). Babies experiencing stunting are at risk of cognitive delays compared with non-stunted infants (8), with approximately 10.8 million stunted infants facing growth delays (9), potentially leading to a 20% reduction in work productivity in adulthood (10).

Various factors influence stunting incidents in children under five years old, as evident in the Framework of Determinant Nutritional Status, including residence, maternal age, marital status, maternal education, maternal occupation, economic status, frequency of antenatal care (ANC), and early initiation of breastfeeding (EIBF) (11). The high incidence of stunting among children residing in rural areas of Bangladesh, around 43% (12), is not unique to Bangladesh, and stunting is prevalent in rural areas due to factors such as early marriage forced by economic constraints, leading to a high dropout rate among mothers (13). Mothers with junior high school education were approximately 1.430 times more likely to have stunted children than those with senior high school education (14). Socioeconomic factors also play a role, as families with lower to middle economic status and large family sizes tend to have limited food availability, which affects the nutritional intake of children under five years old in the household (15). Limited ANC visits and low maternal knowledge about stunting pose risks for giving birth to stunted children, with mothers with fewer than four ANC visits during pregnancy at 1.22 times the risk compared to those with more than four ANC visits (16).

This designation stems from the relatively high proportion of stunting incidents in the Papua region compared with other Indonesian areas. The high incidence of stunting in Papua is attributed to various factors, such as its unique geographical conditions, which differ slightly from other regions, thereby affecting the accessibility of health services in mountainous areas (17). In addition to access to health services, other issues include infrastructure problems and the uneven distribution of healthcare personnel (18). Another contributing factor is the low utilisation of food sourced from animal sources and an appropriate nutritional balance according to the child’s age, which is aggravated by limited access to clean and safe drinking water and adequate sanitation in Papua, where the proportion of access to clean and safe drinking water in 2020 was approximately 88.1% (19). Proper sanitation accounted for approximately 79.5% of cases (20). Previous research in one of the areas in Papua found that pregnant women were primarily young or teenagers (54.9%) with a low education level and an average circumference of < 23.5 cm. Parenting patterns and local cultural practices in maternal and child healthcare that are still upheld in Papuan society also influence children’s health and nutritional status (21). Therefore, this study examined the causes of stunting in Indonesian children.

## Methods

### Study Design and Data Source

Secondary data were obtained from the Indonesian National Nutritional Status Survey conducted in 2022. This nationwide cross-sectional study was conducted by the Indonesian Ministry of Health. The study population comprised all children under five years old in Indonesia’s Papua region. Mothers served as respondents in the survey, whereas children under five years old (< 59 months) served as the analysis unit. A weighted sample of 13,268 Papuan under five years old was collected for the experiment using a multistage cluster random sampling approach. The survey had a response rate of 91.4%.

### Setting

The study was conducted in Indonesia’s Papua region, which comprises two provinces: West Papua and Papua.

### Dependent Variable

The dependent variable in this study was stunting, which measured a child’s nutritional status based on age or height during a certain period. The WHO growth standards produce a height indicator, often known as the Z-score, or height departure from the average size. This study classified stunting into two types: standard and stunting. The upper limit height/age index for the nutritional status category is average (≥ −2.0 standard deviation) and stunting (< −2.0 standard deviation) (1).

### Independent Variables

We investigated 11 control factors: province, type of residence, maternal age, educational level, marital status, employment status, wealth status, ANC during pregnancy, children’s age, sex, and EIBF. We divided the province into western Papua and Papua. Furthermore, the survey divided the residences into urban and rural areas. The determination of the urban-rural criteria refers to Indonesian Statistics.

The study divided mothers into seven age groups: ≥ 19, 20–24, 25–29, 30–34, 35–39, 40–44, and ≥ 45. The marital status groups in this study were married, divorced, or widowed. Maternal employment status encompasses both employed and unemployed. Furthermore, maternal education was divided into four levels: elementary, junior high, senior high, and college.

This study uses the wealth quintile of products to assess a household’s wealth situation. The survey evaluated the number of families and the types of commodities kept in their homes. In addition to population factors, this study utilises a range of goods, including televisions, bicycles, and vehicles, to assess wealth levels. Throughout the evaluation, the survey considered the drinking water supply, bathrooms, and main-level building components. Principal component analysis was used to calculate the scores. The poll sampled 20% of the population, and the pool calculated the national wealth quintiles by adding the household scores for each member. Quintiles were further subdivided into five groups. The survey divided household wealth status into quintiles: 1 (poorest), 2 (poorer), 3 (middle), 4 (richer), and 5 (richest) (22).

Furthermore, ANC during pregnancy included both performed and non-performed ANC. For this study, children were divided into five age groups (in months): 0–11, 12–23, 24–35, 36–47, and 48–59. However, this study distinguished between males and girls. Furthermore, EIBF refers to immediately placing the infant on the mother’s chest or stomach after birth to simplify the baby’s breastfeeding process, which should last at least one hour (23). For EIBF, the response was either “yes” or “no.”

### Data Analysis

Initially, the chi-square test was used. Subsequently, a collinearity test was conducted to determine whether a significant relationship exists among the independent variables, and a binary logistic regression test was then employed. All statistical computations were performed using IBM SPSS Statistics 26. In addition, a distribution map of stunted children was created by the regency/city in Indonesia’s Papua region using ArcGIS 10.3 (ESRI Inc., Redlands, CA, USA). Indonesian Statistics provided a shape file that included administrative border polygons for investigation.

## Results

**Figure 1:**
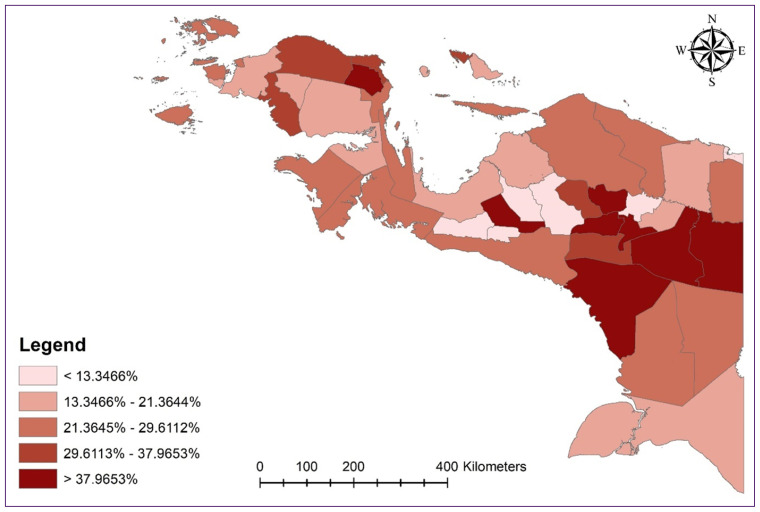
Distribution map of the proportion of stunted toddlers by regency/city in Papua Region, Indonesia Source: Figure visualisation by researchers

The results showed that 29.8% of Papuan under five years old were stunted. Figure 1 displays a distribution map of under five years old stunted children in Indonesia’s Papuan region. Stunting appears to be more common in central mountainous regions, as illustrated in Figure 1.

Table 1 shows the descriptive data on the nutritional condition of Papuan children under five years old in Indonesia. According to province, the prevalence of stunting in children in Papua was higher than that in West Papua. Regarding the type of residence, the ratio of children with stunted growth in rural areas is one-and-a-half times that in urban areas.

Table 1 shows that, based on maternal age, ≥ 19 has the highest stunting prevalence. With regard to maternal education, the higher the educational level, the lower the prevalence of stunted children. Meanwhile, according to maternal employment status, employed mothers had a slightly higher prevalence of stunted children than unemployed mothers. Moreover, according to wealth status, Table 1 indicates that the wealthier the child, the lower the ratio of stunting.

Regarding ANC during pregnancy, Table 1 shows that mothers without ANC had a higher prevalence of stunted toddlers. According to children’s age, those aged 24–35 years had the highest stunting prevalence. Meanwhile, based on children’s sex, boys have a higher stunting prevalence than girls. Furthermore, children without EIBF have a higher stunting ratio.

This study performed the following collinearity tests, which revealed no collinearity among the independent variables. The findings demonstrate that all variable variance inflation factor values are less than 10.00 simultaneously, and all variable average tolerance values are more significant than 0.10. The study discovered no meaningful link between two or more independent variables in the regression model using a multicollinearity test to guide decisions.

Table 2 displays Papuan children’s nutritional status in Indonesia through binary logistic regression findings. The model fitness is: STUNTING = −2.929 + 0.24 PROV + 0.175 PLACE2 + 0.330 MOTHER’S AGE1 + 0.122 MOTHER’S AGE2 − 0.054 MOTHER’S AGE3 − 0.148 MOTHER’S AGE6 + 0.630 EDU1 + 0.254 EDU2 + 0.122 EDU3 + 0.186 MARITAL2 + 0.037 EMPLOYMENT + 0.665 WEALTH1 + 0.513 WEALTH2 + 0.533 WEALTH3 + 0.200 WEALTH4 + 0.139 ANC + 0.992 KID’S AGE2 + 1.046 KID’S AGE3 + 0.868 KID’S AGE4 + 0.775 KID’S AGE5 + 0.276 KID’S GENDER + 0.088 EIBF

Moreover, the binary logistic regression results inform that the Hosmer-Lemeshow *P*-value is < 0.001, and the classification table percentage is 70.9%.

According to the province, Table 2 shows that children under five years old in West Papua are 1.024 times more likely to be stunted than those in Papua (AOR: 1.024, 95% CI: 1.003–1.045). Moreover, regarding the type of residence, children under five years old in rural areas were 1.192 times more likely to be stunted than those in urban areas (AOR: 1.192, 95% CI: 1.167–1.217).

Table 2 shows the four maternal demographic characteristics related to stunting in Papuan children under five years old categorised by age, education, marital status, and employment status. Regarding maternal education, the results showed that the higher the education level, the lower the possibility of having a stunted toddler.

Based on wealth status, Table 2 indicates that all groups were more likely than the richest to have been stunted under five years old. Furthermore, regarding ANC, Papuan mothers without ANC were 1.150 times more likely than those who performed ANC to have been stunted under five years old (AOR: 1.150, 95% CI: 1.116–1.185).

As shown in Table 2, among those under five years old, all age groups were more likely to be stunted than those aged 0–11 months. Regarding the children’s sex, boys were 1.318 times more likely to be stunted than girls (AOR: 1.318, 95% CI: 1.297–1.340). Moreover, according to EIBF, children under five years old were 1.092 times more likely than those with EIBF to be stunted (AOR: 1.092, 95% CI: 1.060–1.124).

## Discussion

According to the Regulation of the President of the Republic of Indonesia Number 72 of 2021, the Sustainable Development Goals target for 2030 is achieved through the implementation of five pillars in the National Strategy for Accelerating Decline Stunting (24):

- increased leadership commitment and vision in ministries/agencies, Regional Government provinces, regional governments, districts and cities, and Village Governments
- increasing behaviour change communication and community empowerment
- increasing convergence of Specific Interventions and Sensitive Intervention in ministries/agencies, Provincial Regional Government, Regional Government district/city, and Village Government
- rising food and nutritional security at the family, individual and community levels
- strengthening and developing data, information, research and innovation systems

Regarding residence, the results showed that children under five years old in West Papua were more likely to be stunted than those in Papua, and those in rural areas were more likely to be stunted than those in urban areas. Papua is one of the most underdeveloped regions in Indonesia. The conditions in the two provinces did not differ significantly. Both are dominated by rural areas that are less affected by development. This situation occurs because mountains with extreme variations loom in the topography. Previous studies have reported that Papua is lagging in development and health (1, 25). The findings of this study align with several previous studies that have reported a higher prevalence of stunting in rural areas compared to urban areas (26–28).

The study also identified four maternal demographic characteristics related to stunting in children under five years old in Papua: age, education, marital status, and employment status. The research results show that young mothers (≥ 19 years) have a higher incidence of stunting. This situation follows research results showing that the risk of stunting in young mothers is at least three times higher than that in adult mothers (29). Educational characteristics show that stunting occurs mainly in mothers with a primary school education. The higher the mother’s education level, the lower the risk of stunting. Mothers with higher education are more likely to engage in practices that can reduce the risk of stunting, such as seeking treatment, practising proper feeding, and engaging in healthy activities during pregnancy (14, 30).

Divorced or separated mothers are at higher risk of giving birth to stunted children. This is because divorced or separated mothers play dual roles as breadwinners. Divorce can also disrupt the operation of a household; therefore, mothers can no longer focus on their children’s health and nutrition (27). These results are not in line with the research in Tanzania, which showed that the proportion of stunted children based on marital status was similar between separated and unmarried mothers (31). The incidence of stunted children among working mothers was higher than that among mothers who did not work. Working mothers are at a higher risk when they provide less household care support and reduce the quality of care for their children (32). Differences in mothers’ employment also determine the incidence of stunting among children. Mothers working in the informal sector can provide extra supervision and care by bringing their children to work, thereby reducing the risk of stunting their children. In contrast to mothers working in the formal sector, caring for their children on weekdays is challenging because they cannot take them to work (33).

Based on wealth status, the results showed that all groups were more likely than the richest to have been stunted before the age of five. The most significant incidence of stunting was observed in the poorest economic status group. A significant relationship was found between the incidence of stunting and financial status. Families with a good economy are able to provide nutrition to children so that their nutritional needs are met, and stunting is avoided. However, families with poor economic conditions are unable to meet their children’s dietary needs and are, therefore, at a significant risk of stunting (34, 35). The wealth index is also a factor found to be significantly associated with neonatal stunting in Ethiopia (36).

Furthermore, Papuan mothers without ANC were more likely than those who performed ANC to have been stunted under the age of five. Mothers who do not regularly provide ANC are at risk of giving birth to stunted children because they do not receive sufficient information regarding health care during pregnancy. Nutritional problems in children during pregnancy are often discovered and treated during ANC; therefore, mothers who rarely attend ANC receive less treatment and health checks related to their pregnancy (37).

According to the children’s age, children aged less than 0–11 months were more likely to be stunted, suggesting that stunted children were within an age range in which the mother’s parenting contributed more to their nutritional status than factors from previous periods or during pregnancy. Other studies have also demonstrated a significant relationship between parenting style and the health level of children under five years old (6–24 months), especially those related to feeding (38). Parents must pay attention to their children’s feeding patterns. Incorrect feeding patterns or conflicts during feeding result in nutritional deficits, growth, and other nutritional issues (39). Additional studies have demonstrated that mothers’ parenting is significantly related to breastfeeding children during the exclusive breastfeeding period, and the beginning of complementary feeding (after six months) is crucial for children’s nutritional status. Mothers’ knowledge, family support, and support from healthcare facilitators are essential in influencing the parenting and feeding patterns of children aged 0–11 months (40).

Child sex may be a critical factor influencing nutritional status. According to this research, regarding under five years old sex, boys were more likely to be stunted than girls. Other studies have found that female children are less likely or have lower odds of being stunted than male children (41, 42). Malnutrition is also more common among boys than among girls (43, 44). Sex awareness is critical for intervening in nutritional issues. There is an endless debate regarding the nutritional status of children in terms of their sex. This could be due to differences in the nutritional requirements of boys and girls; other research has also reported that boys are more likely to be malnourished due to the influence of their environment and parenting style (44). Therefore, interventions to improve the nutritional status of both male and female children should be developed.

Moreover, according to EIBF, this study showed that children under five years old without EIBF were more likely to be stunted than those with EIBF. The World Health Organization recommends the EIBF to reduce mortality, improve feeding behaviour, and provide sufficient nutrients for children. EIBF is an indicator of infant and young feeding that contributes to the nutritional status of children. Previous studies revealed that the probability of wasting and being underweight was lowest among children who received exclusive breastfeeding or EIBF and practised minimum dietary diversity compared to those who did not (45). Another study in Indonesia showed that children without EIBF have a 1.5 higher risk of stunting than children with EIBF (46). Children with EIBF showed a better nutritional status than those without EIBF (47). EIBF proved to improve breastfeeding; therefore, children without EIBF also likely face issues during their breastfeeding period, thus resulting in an unmet need for nutrition, which could cause malnutrition. A prior study in India pointed out that EIBF is protective against several diseases, such as diarrhoea, which could trigger nutritional problems in children (48).

### Strengths and Limitations

This study relied heavily on extensive data analysis to obtain findings for the Papua region. Only the factors listed in the survey were used as additional sources of information for analysis. The conclusions of this study neglected several significant variables examined in other studies that are associated with stunting in children, such as the mother’s height, weight, anaemia, diarrhoea, and agri-food output during pregnancy (30, 49).

However, the quantitative technique employed in this study ignores additional cultural components that persist throughout Indonesia. Numerous other factors, such as the importance of children, foods to avoid, parenting styles, and eating habits, could also impact the associated conclusions (50, 51).

## Conclusion

This study identified 11 factors associated with stunting in Papuan children in Indonesia: province, type of residence, maternal age, education, marital status, employment, wealth, ANC, children’s age, sex, and EIBF. The results indicated that policies for reducing the number of stunted Papuans under five years old in Indonesia should target those who live in West Papua, live in rural areas, are young, have poor education, are single mothers, are unemployed, are poor, do not have ANC, are pregnant, have a son, and do not receive EIBF.

The study showed that Papuan Indonesia faces significant obstacles in controlling stunting. This study implies that those under five years old in the West Papua region have a higher risk of exposure to the causes of stunting compared to those in Papua. This exposure causes reduced nutritional intake during the child’s growth period. Those under five years old who live in rural areas also experience obstacles to getting into situations that support toddler growth. These obstacles can arise from the topography of the Papuan Indonesian region, which varies significantly from highlands to lowlands. Another implication is that young mothers in Papua have challenges in meeting their children’s growing needs. These challenges are doubled if the mother has a low level of education, is unemployed, poor, and does not have a partner. Pregnant women who do not receive ANC and, when giving birth, do not perform EIBF have the potential to have obstacles in providing care to support the child’s growth. Toddler boys require more attention from their parents because they are susceptible to growth disorders compared to girls.

The recommendation from this study is that a stunting reduction policy needs to be prioritised in the West Papua region. This policy must be easily accessible to children under five years old in rural areas. The socialisation of the importance of higher formal education and the delay of early marriage must also be done intensively. Health workers must continuously supervise ANC and EIBF. High-risk groups, including the unemployed, poor, and single mothers, need empowerment programmes in the form of relevant life skills. We can carry out the empowerment programme through cross-programme and cross-sectoral collaboration.

## Availability of Data and Materials

The author cannot publicly share the data because a third party and the Ministry of Health of the Republic of Indonesia, who owns the data, do not have permission to share it. The 2022 Indonesian National Nutrition Status Survey dataset is available online at https://layanandata.kemkes.go.id/ for researchers who meet the criteria for access to confidential data.

## Figures and Tables

**Table 1:** Descriptive statistic of nutritional status among Papuan under five years old in Indonesia (*n* = 13,268)

Variables	Nutritional status		*P*-value	Degree of freedom

	Normal (*n* = 9,530)	Stunted (*n* = 3,738)		
Province			< 0.001	1
West Papua	73.3 %	26.7 %		
Papua	69.2 %	30.8 %		

The type of residence			< 0.001	1
Urban	78.1 %	21.9 %		
Rural	65.2 %	34.8 %		

Maternal age (in years)			< 0.001	6
≥ 19	65.0 %	35.0 %		
20–24	66.7 %	33.3 %		
25–29	70.4 %	29.6 %		
30–34	70.4 %	29.6 %		
35–39	71.8 %	28.2 %		
40–44	73.5 %	26.5 %		
≥ 45	67.2 %	32.8 %		

Maternal education			< 0.001	3
Primary school	59.5 %	40.5 %		
Junior high school	71.0 %	29.0 %		
Senior high school	77.2 %	22.8 %		
College	82.4 %	17.6 %		

Maternal marital status			< 0.001	1
Married	70.3 %	29.7 %		
Divorced/widowed	67.0 %	33.0 %		

Maternal employment status			< 0.001	1
Unemployed	70.4 %	29.6 %		
Employed	69.9 %	30.1 %		

Wealth status			< 0.001	4
Poorest	62.6 %	37.4 %		
Poorer	72.1 %	27.9 %		
Middle	73.8 %	26.2 %		
Richer	80.3 %	19.7 %		
Richest	84.5 %	15.5 %		

Perform ANC during pregnancy			< 0.001	1
No	66.6 %	33.4 %		
Yes	79.7 %	20.3 %		

Under five years old’s age (in months)			< 0.001	4
0–11	84.5 %	15.5 %		
12–23	67.0 %	33.0 %		
24–35	63.8 %	36.2 %		
36–47	66.7 %	33.3 %		
48–59	69.2 %	30.8 %		

Under five years old’s sex			< 0.001	1
Boy	67.2 %	32.8 %		
Girl	73.2 %	26.8 %		

Early initiation of breastfeeding			< 0.001	1
No	68.1 %	31.9 %		
Yes	79.4 %	20.6 %		

**Table 2:** Binary logistic regression of nutritional status of Papuan under five years old in Indonesia (*n* = 13,268)

Predictors	Stunting			

	AOR	95% CI		*P*-value

		Lower bound	Upper bound	
Province: Papua (ref.)	–	–	–	–
Province: West Papua	1.024	1.003	1.045	*0.023
Residence: Urban (ref.)	–	–	–	–
Residence: Rural	1.192	1.167	1.217	**< 0.001
Maternal age: ≥ 45 (ref.)	–	–	–	–
Maternal age: ≥ 19	1.390	1.295	1.493	**< 0.001
Maternal age: 20–24	1.130	1.075	1.187	**< 0.001
Maternal age: 25–29	0.947	0.904	0.993	*0.024
Maternal age: 30–34	0.975	0.930	10.021	0.280
Maternal age: 35–39	0.978	0.932	10.027	0.371
Maternal age: 40–44	0.863	0.819	0.909	**< 0.001
Maternal education: College (ref.)	–	–	–	–
Maternal education: Primary school	1.877	1.809	1.948	**< 0.001
Maternal education: Junior high school	1.289	1.241	1.339	**< 0.001
Maternal education: Senior high school	1.130	1.092	1.170	**< 0.001
Maternal marital: Married (ref.)	–	–	–	–
Maternal marital: Divorced/widowed	1.204	1.143	1.269	**< 0.001
Maternal employment: Employed (ref.)	–	–	–	–
Maternal employment: Unemployed	1.038	1.020	1.056	**< 0.001
Wealth: Richest (ref.)	–	–	–	–
Wealth: Poorest	1.945	1.875	2.018	**< 0.001
Wealth: Poorer	1.671	1.608	1.736	**< 0.001
Wealth: Middle	1.704	1.635	1.776	**< 0.001
Wealth: Richer	1.221	1.175	1.269	**< 0.001
Perform ANC during pregnancy: Yes (ref.)	–	–	–	–
Perform ANC during pregnancy: No	1.150	1.116	1.185	**< 0.001
Under five years old’s age: 0–11 (ref.)	–	–	–	–
Under five years old’s age: 12–23	2.698	2.623	2.774	**< 0.001
Under five years old’s age: 24–35	2.846	2.753	2.941	**< 0.001
Under five years old’s age: 36–47	2.383	2.305	2.463	**< 0.001
Under five years old’s age: 48–59	2.171	2.096	2.249	**< 0.001
Under two years old’s sex: Girl (ref.)	–	–	–	–
Under two years old’s sex: Boy	1.318	1.297	1.340	**< 0.001
Early initiation of breastfeeding: Yes (ref.)	–	–	–	–
Early initiation of breastfeeding: No	1.092	1.060	1.124	**< 0.001

AOR = adjusted odds ratio; CI = confidence interval;

* *P* < 0.050;

** *P* < 0.001

## References

[1] Wulandari RD, Laksono AD, Kusrini I, Tahangnacca M (2022). The targets for stunting prevention policies in Papua, Indonesia: what mothers’ characteristics matter?. Nutrients.

[2] World Health Organization (2019). WHA global nutrition targets 2025: stunting policy brief [Internet].

[3] World Health Organization (2023). Stunting prevalence among children under 5 years of age (%) (model-based estimates) [Internet].

[4] Ministry of National Development Planning/Bappenas (2017). Roadmap for Sustainable Development Goals in Indonesia [Peta Jalan Sustainable Development Goals di Indonesia].

[5] UNICEF, World Health Organization World Bank Group (2021). Levels and trends in child malnutrition.

[6] The Ministry of Health of The Republic of Indonesia (2023). The results of 2022 Indonesian nutritional status survey [Hasil survei status gizi Indonesia 2022] [Internet].

[7] Soliman A, De Sanctis V, Alaaraj N, Ahmed S, Alyafei F, Hamed N (2021). Early and long-term consequences of nutritional stunting: from childhood to adulthood. Acta Biomed.

[8] Mustakim MRD, Irwanto, Irawan R, Irmawati M, Setyoboedi B (2022). Impact of stunting on development of children between 1–3 years of age. Ethiop J Health Sci.

[9] Danaei G, Andrews KG, Sudfeld CR, Mccoy C, Peet E, Sania A (2016). Risk factors for childhood stunting in 137 developing countries: a comparative risk assessment analysis at global, regional, and country levels. PLoS Med.

[10] Suryana EA, Azis M (2023). The potential of economic loss due to stunting in Indonesia. J Ekon Kesehat Indones.

[11] UNICEF (2020). UNICEF conceptual framework on maternal and child nutrition [Internet].

[12] Sarma H, Khan JR, Asaduzzaman M, Uddin F, Tarannum S, Hasan MM (2017). Factors influencing the prevalence of stunting among children aged below five years in Bangladesh. Food Nutr Bull.

[13] Tambing Y, Fatiah MS, Apriyana I (2023). Perbedaan usia pernikahan anak pada perempuan pernah kawin usia 15 – 24 tahun di perdesaan dan perkotaan Indonesia. J Bidan Cerdas.

[14] Laksono AD, Wulandari RD, Wisnuwardani RW, Amaliah N (2022). Stunting among children under two years in Indonesia: does maternal education matter?. PLoS One.

[15] Sumarmi S (2020). Determinan sosial penanggulangan stunting: riset aksi partisipatif desa sehat berdaya fokus penanggulangan stunting [Social determinants of stunting countermeasures: participatory action research in healthy villages empowered by stunting countermeasures].

[16] Titaley CR, Ariawan I, Hapsari D, Muasyaroh A, Dibley MJ (2019). Determinants of the stunting of children under two years old in Indonesia: a multilevel analysis of the 2013 Indonesia basic health survey. Nutrients.

[17] Wulandari RD, Laksono AD, Matahari R, Rohmah N, Krismawati H (2021). Kinerja pelayanan kesehatan ibu dan anak di Papua tahun 2018: apakah input tenaga bidan dan dokter berpengaruh? [Performance of maternal and child health services in Papua in 2018: does the input of midwives and doctors have an effect?]. Bul Penelit Sist Kesehat.

[18] Sumule AI, Suratman E, Indra, Tuerah N, Reba WH (2023). Kajian peningkatan pelayanan kesehatan berkualitas pada fasilitas pelayanan kesehatan di Provinsi Papua dan Papua Barat [Study of improving quality health services in health service facilities in Papua and West Papua Provinces].

[19] Papua Province, World Food Programme, Papua Food Security Council (2016). Peta ketahanan dan kerentanan pangan Papua [Map of Papua’s food security and vulnerability].

[20] Indonesian Ministry of State Secretaries (2022). Dashboard pemantauan terpadu percepatan pencegahan stunting [Integrated monitoring dashboard to accelerate stunting prevention] [Internet].

[21] Laksono AD, Wulandari RD (2019). “Children are assets”: meta synthesis of child values in the Lani and Acehnese [“Anak adalah aset”: meta sintesis nilai anak pada suku Lani dan suku Aceh]. J Kesehat Reproduksi.

[22] Wulandari RD, Laksono AD, Prasetyo YB, Nandini N (2022). Socioeconomic disparities in hospital utilization among female workers in Indonesia: a cross-sectional study. J Prim Care Community Health.

[23] Dubik SD, Amegah KE (2021). Prevalence and determinants of early initiation of breastfeeding (EIBF) and prelacteal feeding in Northern Ghana: a cross-sectional survey. PLoS One.

[24] President of the Republic of Indonesia (2021). Peraturan presiden nomor 72 tahun 2021 tentang percepatan penurunan stunting [Presidential regulation number 72 of 2021 concerning the acceleration of reducing stunting]. [Internet].

[25] Laksono AD, Wulandari RD (2021). Pantangan makanan pada suku Muyu di Papua [The food taboo of the Muyu tribe in Papua]. Amerta Nutr.

[26] Ipa M, Yuliasih Y, Astuti EP, Laksono AD, Ridwan W (2023). Stakeholders’ role in the implementation of stunting management policies in Garut Regency. Indones J Heal Adm.

[27] Laksono AD, Sukoco NEW, Rachmawati T, Wulandari RD (2022). Factors related to stunting incidence in toddlers with working mothers in Indonesia. Int J Environ Res Public Health.

[28] Kusrini I, Laksono AD (2020). Regional disparities of stunted toddler in Indonesia. Indian J Forensic Med Toxicol.

[29] Wemakor A, Garti H, Azongo T, Garti H, Atosona A (2018). Young maternal age is a risk factor for child undernutrition in Tamale Metropolis, Ghana. BMC Res Notes.

[30] Amaha ND, Woldeamanuel BT (2021). Maternal factors associated with moderate and severe stunting in Ethiopian children: analysis of some environmental factors based on 2016 Demographic Health Survey. Nutr J.

[31] Mtongwa RH, Festo C, Elisaria E (2021). A comparative analysis of determinants of low birth weight and stunting among under five children of adolescent and non-adolescent mothers using 2015/16 Tanzania Demographic and Health Survey (TDHS). BMC Nutr.

[32] Win H, Shafique S, Mizan S, Wallenborn J, Probst-Hensch N, Fink G (2022). Association between mother’s work status and child stunting in urban slums: a cross-sectional assessment of 346 child-mother dyads in Dhaka, Bangladesh (2020). Arch Public Health.

[33] Hosen MZ (2023). Impact of maternal employment on children malnutrition status in Bangladesh: an empirical analysis. J Soc Econ Dev.

[34] Kumar P, Rashmi R, Muhammad T, Srivastava S (2021). Factors contributing to the reduction in childhood stunting in Bangladesh: a pooled data analysis from the Bangladesh demographic and health surveys of 2004 and 2017–18. BMC Public Health.

[35] Budiarto W, Wulandari RD, Rohmah N, Laksono AD (2021). Ecological relationship between poverty and nutritional status of toddler in Indonesia in 2018. Indian J Forensic Med Toxicol.

[36] Tola G, Kassa A, Getu M, Dibaba B, Neggesse S (2023). Prevalence of stunting and associated factors among neonates in Shebadino Woreda, Sidama Region South Ethiopia; a community-based cross-sectional study 2022. BMC Pediatr.

[37] Tesfaye A, Egata G (2022). Stunting and associated factors among children aged 6–59 months from productive safety net program beneficiary and non-beneficiary households in Meta District, East Hararghe Zone, Eastern Ethiopia: a comparative cross-sectional study. J Health Popul Nutr.

[38] Rafa M (2022). Hubungan sikap, dukungan keluarga dan pola asuh terhadap status gizi balita usia 6–24 bulan di Puskesmas Menes [Relationship between attitude, family support and parenting patterns on the nutritional status of toddlers age 6–24 months at the Menes Community Health Center]. Indones Sch J Nurs Midwifery Sci.

[39] Milano K, Chatoor I, Kerzner B (2019). A functional approach to feeding difficulties in children. Curr Gastroenterol Rep.

[40] Athavale P, Hoeft K, Dalal RM, Bondre AP, Mukherjee P, Sokal-Gutierrez K (2020). A qualitative assessment of barriers and facilitators to implementing recommended infant nutrition practices in Mumbai, India. J Health Popul Nutr.

[41] Sultana P, Rahman MM, Akter J (2019). Correlates of stunting among under-five children in Bangladesh: a multilevel approach. BMC Nutr.

[42] Chowdhury TR, Chakrabarty S, Rakib M, Afrin S, Saltmarsh S, Winn S (2020). Factors associated with stunting and wasting in children under 2 years in Bangladesh. Heliyon.

[43] Thurstans S, Opondo C, Seal A, Wells J, Khara T, Dolan C (2020). Boys are more likely to be undernourished than girls: a systematic review and meta-analysis of sex differences in undernutrition. BMJ Glob Health.

[44] Mkhize M, Sibanda M (2020). A Review of selected studies on the factors associated with the nutrition status of children under the age of five years in South Africa. Int J Environ Res Public Health.

[45] Satapathy D, Karmee N, Sahoo S, Patro S, Pandit D (2021). Effect of feeding practices on nutritional status of infant and young children residing in urban slums of Berhampur: a decision tree approach. Indian J Public Health.

[46] Permanasari Y, Saptarini I, Amalia N, Aditianti A, Safitri A, Nurhidayati N (2021). Faktor determinan balita stunting pada desa lokus dan non lokus di 13 kabupaten lokus stunting di Indonesia tahun 2019 [Determinant factors for stunting of toddlers in locus and non-locus villages in 13 stunting locus districts in Indonesia in 2019]. J Nutr Food Res.

[47] Aryanti PM, Widarti IGAA, Sukraniti DP (2020). Inisiasi menyusui dini dan usia penyapihan dengan status gizi anak usia 6-24 bulan. JIG J Nutr Sci.

[48] Dhami M, Ogbo F, Diallo T, Agho K (2020). Regional analysis of associations between infant and young child feeding practices and diarrhoea in Indian children. Int J Environ Res Public Health.

[49] Castro-Bedriñana J, Chirinos-Peinado D, De La Cruz-Calderón G (2021). Predictive model of stunting in the Central Andean region of Peru based on socioeconomic and agri-food determinants. Public Health Pract.

[50] Laksono AD (2015). Anyiman: studi etnografi makanan suku Muyu.

[51] Kusrini I, Ipa M, Laksono AD (2019). “Is it true that the child is king?”: qualitative study of factors related to nutritional status of children in West Lombok, Indonesia. Indian J Public Health Res Dev.

